# Malignant Bone and Soft Tissue Lesions of the Foot

**DOI:** 10.3390/jcm12083038

**Published:** 2023-04-21

**Authors:** Andrea Angelini, Carlo Biz, Mariachiara Cerchiaro, Valentina Longhi, Pietro Ruggieri

**Affiliations:** Department of Orthopedics and Orthopedic Oncology, University of Padova, 35128 Padova, Italypietro.ruggieri@unipd.it (P.R.)

**Keywords:** foot tumors, synovial sarcoma, malignant tumors, extremities, differential diagnosis

## Abstract

Malignant tumors of the foot are rare pathologies that can involve the skin, soft tissue, or bone. Due to their rarity, they are often misdiagnosed, resulting in inadequate excision and poor outcomes. A correct approach with a careful examination and radiological study, followed by a properly performed biopsy, is thus mandatory to avoid these pitfalls. The present article reviews the most common malignant bone and soft tissue lesions of the foot region, discussing their clinicopathological presentation, imaging features, and current concepts in treatment.

## 1. Introduction

Malignant foot and ankle tumors are rare lesions, contributing to approximately 3% of all cases of bone tumors and 5% of malignant soft-tissue tumors [1,2,3,4,5,6,7,8,9]. These entities should always be considered in the differential diagnosis when evaluating a foot lesion. Indeed, they can easily be overlooked due to their rarity and unspecific symptoms, which often cannot be used to discriminate between benign and malignant lesions. Furthermore, patients tend to neglect them, seeking medical attention only in the presence of pain or significant swelling, thus commonly resulting in diagnostic delay and improper treatment.

In this paper, the most common malignant bone and soft-tissue tumors of the foot are presented, discussing their epidemiology and presentation, their clinical and instrumental diagnosis, and principles of treatment in order to enhance the knowledge of these pathologies and favor the correct diagnostic and therapeutic strategy.

## 2. Evaluation

The first approach includes a careful history, to obtain information about a patient’s age, how the lesion developed and was noted, the rate of growth in terms of aggressiveness, and other symptoms such as pain, numbness, fever, or weight loss. Pain characteristics, i.e., type, severity, and worsening during activity or during rest, need to be investigated. Any history of exposure to sun, radiation, chemicals, or pollutants should be recorded. Concomitant or previous diseases, particularly malignancies, should be investigated. Any data about prior biopsies or surgery should be examined [10].

A careful physical examination is mandatory. Inspection should exclude any skin lesions, discolorations, or visible lumps. On palpation, any mass should be characterized in terms of location, size, shape, consistency, mobility, and tenderness. Any palpable lymph nodes should be recorded. A thorough neurovascular examination should be included.

Laboratory tests can be helpful in guiding the diagnostic algorithm: in some tumor histotypes, an increase in the erythrocyte sedimentation rate can be observed (Ewing’s sarcoma or hematologic neoplasms), while others specifically modify alkaline phosphatase (osteosarcoma).

Whereas there are no doubts that radiographs and ultrasound represent the first imaging approach for bone and soft tissue lesions, respectively, there is no international uniform method of staging. X-rays represent an essential first step. In bone tumors, they show the location of the lesion (diaphyseal, metaphyseal, epiphyseal, central, eccentric, intracortical), its aspect (lytic, sclerosing, mixed), the pattern of bone destruction, i.e., geographical vs moth-eaten appearance, and the presence of any calcifications, cortical thinning or destruction, or periosteal reaction. The risk for pathological fractures can also be estimated from X-rays [10]. In soft tissue lesions, X-rays are useful to highlight the presence of soft tissue calcifications, but computed tomography (CT) is more effective for further assessing bony details, allowing the study of the cortical bone and periosteal reactions and small calcifications. Ultrasound imaging can be a first approach for soft tissue lesions, as it can differentiate the mass in relation to its content and the type of tissue of origin (adipose, vascular, or nervous); however, it is highly dependent on operator experience. Magnetic resonance (MR) imaging is the reference method in the study of soft-tissue sarcomas, above all for its ability to differentiate the type of tissue on a contrastographic basis and to define the extent of the disease between bone and soft tissue, whereas in bone sarcomas it provides extensive details about tumor extent, components, anatomical relationship with adjacent structures, and bony edema. What is important when dealing with foot soft-tissue tumors, and therefore unique to the foot, is the need to correctly recognize foot compartments. Sands et al. classified nine functional compartments considering the diagnosis and treatment of compartment syndrome in the foot [11]. From an oncological point of view, the foot’s fasciae cannot be considered a real anatomical barrier against tumor growth. Mercuri reported a specific classification of “oncologic” compartments in the foot based on anatomic tissue: bony, joint, and muscle [12]. The anatomic areas are completely different between the fore-, middle, and rearfoot. Whereas the forefoot can be divided into separate compartments such as individual rays, the hindfoot is oncologically “unsafe” with discontinuous fascial planes. Other subcutaneous and perivascular areas are considered fully extra-compartmental: the dorsum pedis, the subcutaneous space, and areas close to neuromuscular bundles [13].

In cases in which malignancy is highly suspected, or after a histologic confirmation of a malignant tumor, a chest CT scan and a bone scan should be performed to assess any metastatic spread. Other exams such as whole-body PET-CT and abdominal/pelvic CT scans should be considered in specific settings and histotypes [14,15].

Biopsy is an important step of staging, but not necessarily the last. Performing a biopsy before completing the staging avoids unnecessary second-level systemic imaging tests in benign tumors (which are 200 times more frequent). Staging prior to receiving histopathological findings is indicated if there is almost no doubt of malignancy and there is no time to waste to start neoadjuvant therapy (e.g., in osteosarcoma or Ewing’s sarcoma). An adequate biopsy provides representative and viable tissue to make a correct diagnosis. The type of biopsy performed depends on the clinical situation (type of tumor, location), the surgeon’s skills, and the pathologist’s interpretive experience. A core needle biopsy is the gold standard for sampling that maintains tissue architecture, with a low rate of contamination and a minimally invasive approach [16,17]. It can be imaging-guided, usually by ultrasound, X-ray, or CT scan. An open incisional biopsy, which involves removing a tissue sample from within the tumor, is indicated when a needle biopsy is not technically feasible (due to proximity to skin or bone) or in previous nondiagnostic biopsies. Excisional biopsy is an open surgical procedure of tumor excision with surrounding normal tissue; currently, the authors have only performed an excisional biopsy for tumors too small to be approached by a core needle biopsy and which can be excised with safe wide margins. It is clear that some pathognomonic benign lesions can be easily detected and treated with open excisional surgery by an experienced musculoskeletal tumor surgeon, but this message is potentially dangerous. In fact, it is the common experience of specialized centers to manage patients who have been erroneously treated elsewhere with the inadequate excision of malignant tumors, especially in feet and hands [18,19]. Accurate planning of the biopsy site is of paramount importance in order to minimize the risk of the contamination of adjacent tissues and the dissemination of the disease, as a poorly placed biopsy can preclude limb-salvage surgery [20].

## 3. Malignant Bone Tumors

### 3.1. Osteosarcoma

Osteosarcoma is rarely observed in the foot (hindfoot bones and metatarsals) and is well described in small series or reports, with an incidence ranging from 0.5 to 1.3% of all osteosarcomas [21]. In the foot, osteosarcoma tends to have a later presentation than in other regions, with a median age of 32 years; it shows a slight male predominance [22]. Pain is the main symptom, often associated with swelling [23].

In the family of osteosarcomas, some subcategories may present osteolytic behavior (i.e., telangiectatic osteosarcoma), predisposing pathological fracture as an onset symptom. In osteoblastic variants, the osteoid deposition may calcify, causing the typical intralesional hyperdensity changes and periosteal reaction with Codman’s triangle on imaging. MRI allows for the easy identification of bony and intramedullary extension and the invasion of surrounding soft tissue [24].

Neoadjuvant chemotherapy regimens and surgery for systemic and local control, respectively, are the standard management for osteosarcoma. Four drugs are classically used in each cycle of chemotherapy (doxorubicin, cisplatin, methotrexate, and cyclophosphamide), dividing the treatment into administrations before and after surgery. Surgery involves the removal of the primary tumor and the subsequent reconstruction procedure if limb salvage is possible. Enneking et al. clearly classified resection margins as intralesional, marginal, wide, and radical [25], but some aspects have changed during recent years, especially with the improvement of chemotherapy efficacy [26,27]. Based on the anatomic concepts of compartments in the foot, adequate oncologic surgery is possible only with wide or radical margins. Approximately 25% of osteosarcoma patients have lung metastases at diagnosis, whereas 60–70% developed metastases within 5 years of follow-up from primary treatment [28]. Local recurrence and distant metastasis affect one-third of the patients within 2 years after surgery and represent the most important prognostic factor for overall survival [28]. Radiotherapy is not effective [29]. Metastases can also appear in other areas besides the lung [30]. Metachronous metastases are quite common, with an incidence of approximately 50–60%, whereas 20% of cases develop synchronous metastases [30].

### 3.2. Chondrosarcoma

Chondrosarcomas (CHS) are the second most common primary bone tumors, characterized by the ability of tumor cells to produce a cartilage matrix. More than 90% of cases are conventional CHS [31]. Variant subtypes, significantly less common, include mesenchymal, clear cell, and extraskeletal mixoid types. The dedifferentiated CHS variant presents, simultaneously, areas of low-grade conventional CHS and a part degenerating into a highly aggressive sarcomatous form, with characteristics of osteosarcoma, fibrosarcoma, or undifferentiated pleomorphic sarcoma [32,33].

Whereas the pelvic bones, humerus, femur, and chest wall are the most common sites involved, the small bones of the feet are rarely affected (<1% of all cases reported cases). The involvement of toes is extremely rare [34]. These tumors affect adult and elderly patients, mainly between the fourth and sixth decades of life. They may arise primarily or secondarily (malignant degeneration of an enchondroma or an osteochondroma) [35].

Unlike other chondrosarcomas, which tend to be confined in the metadiaphysis, in the feet, these lesions involve the majority of the length of the bone. Characteristic features include endosteal scalloping greater than two-thirds of the cortex or soft tissue involvement. MRI shows intermediate to high signal intensity on T1-weighted imaging and hyperintensity on fluid-sensitive sequences. In the small tubular bones of the hands and feet, the histologic threshold for the diagnosis of CHS is much higher than that for lesions elsewhere in the body [36]. CHS are graded into three categories according to Evans et al. [37]. Histologic parameters for grading include cellularity, binucleated cells per high power field, cellular distribution, nuclear pleomorphism, bone formation, differentiation (percentage chondroid, mucoid, and myxoid differentiation), the presence of calcification, and cortical destruction [38].

With regard to grade 1 chondrosarcomas, the current literature has drastically changed the approach compared to the past. This tumor type is also referred to as an atypical chondromatous tumor (ACT) to avoid rigorous overtreatment. Although the histological appearance is similar to benign cartilage lesions, a multidisciplinary evaluation and the interpretation of clinical, imaging, and histological features are required to combine radiographic interpretation and clinical evaluation with histological findings to obtain an accurate preoperative diagnosis of grade 1 central chondrosarcoma.

**Figure 1:**
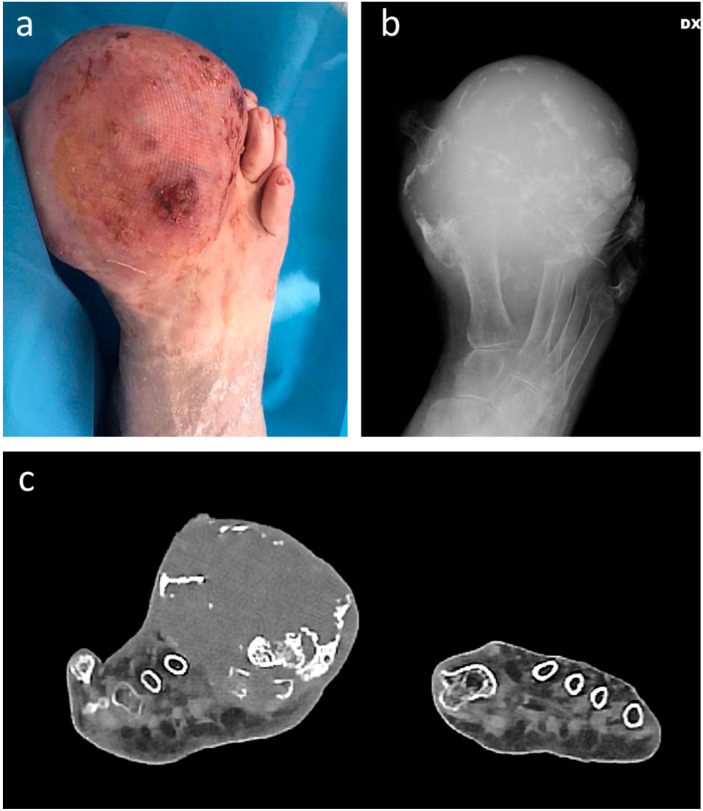
Clinical (**a**), plain radiograph (**b**), and coronal CT scan (**c**) appearance of gr. 2 chondrosarcoma in a 95-year-old female patient, appearing as swelling gradually increased over the last 15 years. A below-knee amputation was performed.

The histological grade represents an independent prognostic factor for the survival of patients with CHS. However, the differentiation between enchondroma and ACT is the focus of extensive discussion in the literature [39]. Surgery is the mainstay of treatment, as most chondrosarcoma subtypes are resistant to conventional chemo-/radiotherapy protocols (Figure 1). The objective of surgery is local control (avoiding excision with contamination of the margins and therefore the potential risk of distant metastases) and the restoration of the residual functionality of the limb. Intralesional resection, instead of resection, can be regarded as a safe and function-preserving treatment for ACT [40].

### 3.3. Ewing Sarcoma

**Figure 2:**
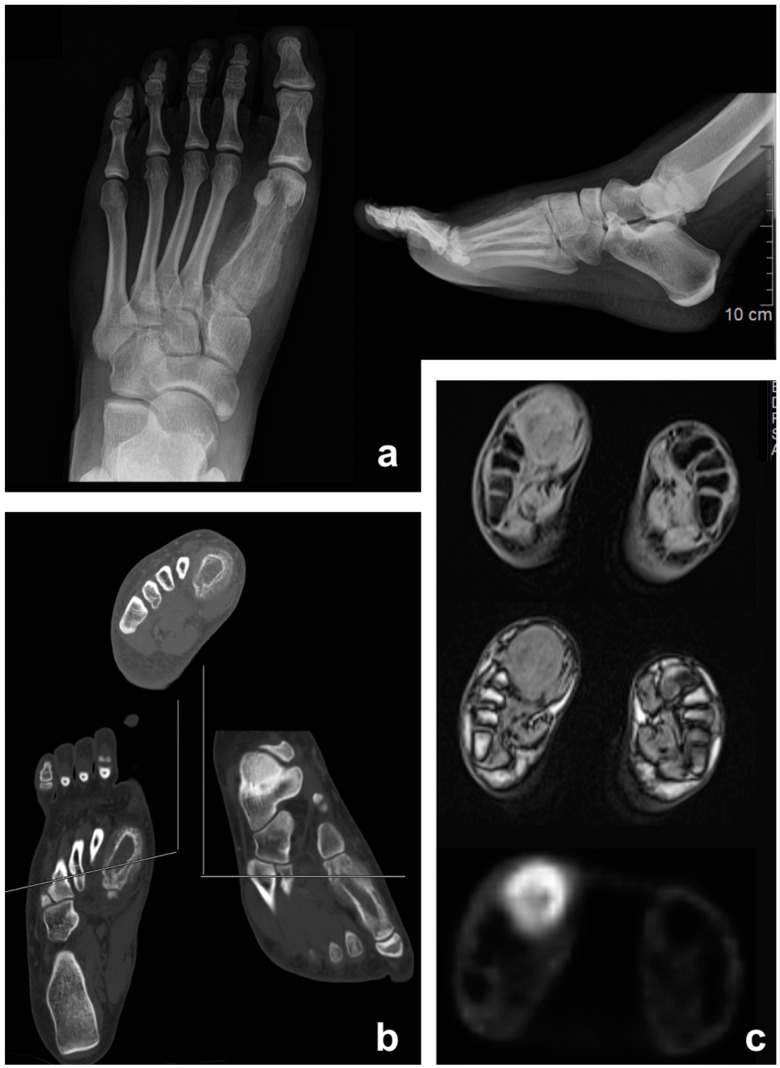
Ewing sarcoma of the foot (first metatarsal) in a 17-year-old female patient: Appearance on antero-posterior X-ray (**a**), CT scan (**b**), and PET/MRI (**c**).

Ewing sarcoma (ES) is usually observed in males (O.R. of 1.5) in the first two decades of life. ES is the third most frequent primary bone tumor affecting long bones (47%, mainly in the diaphysis), the pelvis, the chest wall, and the spine, even if it can rarely originate from soft tissue. The small bones of the foot are rarely involved, ranging from 3% to 5% of the reported cases [41]. Patients with ES of the foot commonly have a few months’ history of painful swelling, which can impair ambulation and shoe-wearing. Ewing sarcoma can be misdiagnosed as osteomyelitis, resulting in delayed diagnosis. Pathological fractures may affect the involved bone at initial presentation or during/after treatment [42]. Radiographs show a lytic lesion with a permeative appearance through the cortex, with a large extra-osseous component. As for OS, MR is the most appropriate imaging test for assessing tumor volume and guiding surgical and chemo-/radiotherapy treatment [43] (Figure 2).

On macroscopic evaluation, the tumor appears as a multilobulated mass with extensive necrotic and hemorrhagic areas, while on microscopic evaluation, the typical carpet of small, round monomorphic cells with vesicular nuclei with finely dispersed chromatin is present and scant cytoplasm. Staging and response to chemotherapy are the most important prognostic factors for ES [44]. Synchronous metastases at diagnosis are present in about 15–30% of patients and are related to a poorer prognosis [45].

The ES treatment strategy includes a multidrug chemotherapy approach and local control, which can be performed with surgery, radiotherapy, or both. Despite the anatomical difficulties, local control is of primary importance, considering the reduced survival rates in case of metastatic spread or local recurrence of the disease [46].

### 3.4. Acrometastases

**Figure 3:**
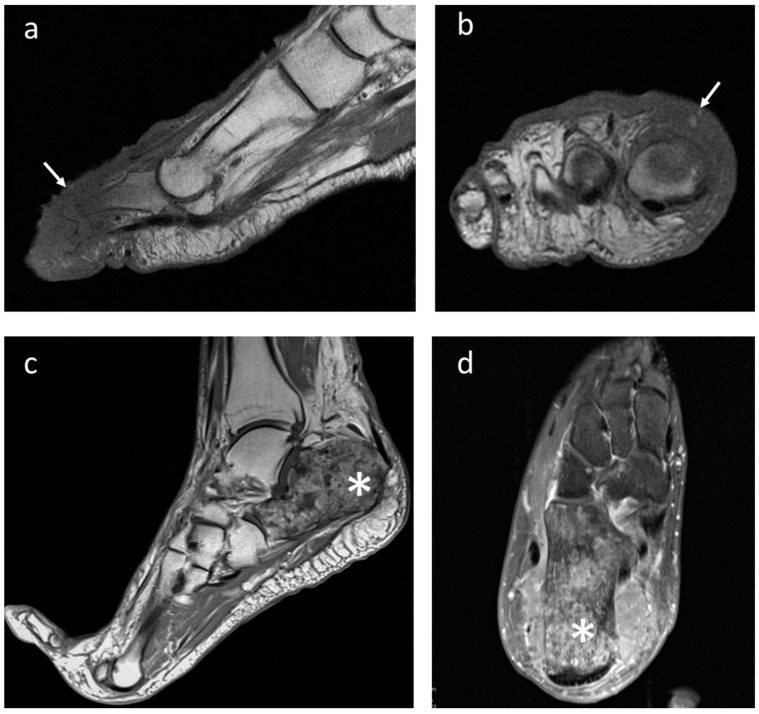
Right forefoot metastasis in a female patient with anaplastic large cell lymphoma (white arrows). Imaging appearance at MRI in (**a**) sagittal and (**b**) coronal projection; (**c**) metastasis from melanoma showing a full necrotic appearance of the calcaneus (asterisk) on T1-weighted sagittal and (**d**) transverse T2-weighted sequences.

Metastases affecting the bones distally to the bone or elbow are called “acrometastases” and are rare, with an incidence of 7% and a median age of presentation of about 60 years old [47]. Moving to the distal phalanges, the incidence drops to 0.05%. Breast and lung carcinoma are the main primary tumors. Since they are usually found in widespread cancer disease, hand and foot metastases are indicators of poor prognosis. Generally, the mean life expectancy after acrometastases diagnosis is about 6 months [48] (Figure 3).

Although it is not yet clearly understood, hematogenous dissemination through the microcirculation of the extremities seems to be the most accredited hypothesis. In fact, it would explain the higher prevalence of lung tumors because tumor cells have immediate access to the systemic circulation through the left atrium and ventricle. Symptoms include tenderness, pain, functional impairment, erythema, heat, and swelling [49]. Often, the primary tumor is unknown and the acrometastasis is the presenting symptom. Appropriate local imaging (X-ray, MRI, and CT scan) is the first step of the diagnostic protocol, but a tumor-tissue biopsy is needed to establish the histological diagnosis [16,17,50].

Radical surgery is the treatment of choice for acrometastases depending on prognosis and the risk of tumor ulceration, and amputation is the preferred option in more distal lesions. In all patients affected by bone metastases from carcinoma, systemic chemotherapy represents the only valid weapon for prognostic purposes, but surgery and radiotherapy can be useful in maintaining a good quality of life and limb function [51]. The prognosis is unfavorable even after appropriate treatment for the age of the patient and widespread disease [52].

## 4. Soft-Tissue Sarcomas

Soft-tissue sarcomas (STSs) of the distal extremities account for less than 5% of sarcomas. Synovial sarcoma, epithelioid sarcoma, and clear-cell sarcoma are the most frequently reported malignancies in the foot and hand [1,53,54]. More rarely, rhabdomyosarcoma, leiomyosarcoma, liposarcoma, and fibrosarcoma can be observed [1,54,55]. STS of the foot may have clinical characteristics similar to benign lesions: small size, slow growth, and painlessness. Surely the reduced presence of muscle tissue in the foot and ankle allows for the visualization of tumor masses of smaller dimensions than what occurs in the typical sites at the root of the limbs [56].

Because of its rarity, the management of STS in the hand and foot is not well established, with a 5-year survival rate ranging from 67% to 80% [57]. The so-called whoops surgery (when a mass, which subsequently turns out to be an STS, is surgically removed with inadequate margins) occurs frequently in the foot, placing patients at a high risk of local recurrence and poor prognosis. These lesions should be treated at a tumor center, with the aim of obtaining adequate margins during the first surgery to remove the disease. Adjuvant chemotherapy for STS remains controversial, especially in the foot and ankle, whereas radiotherapy may be used to eradicate potential residual microscopic disease [55], although its use remains under debate because of the risk of wound complications and soft tissue fibrosis [58]. A demolitive surgery is still used in selected cases, where it is not possible to preserve the extremity of the limb in conditions of adequate vascularization and functionality [59]. However, multidisciplinary approaches such as orthoplasty play a growing role in limb-salvage surgery [60,61].

### 4.1. Melanoma

**Figure 4:**
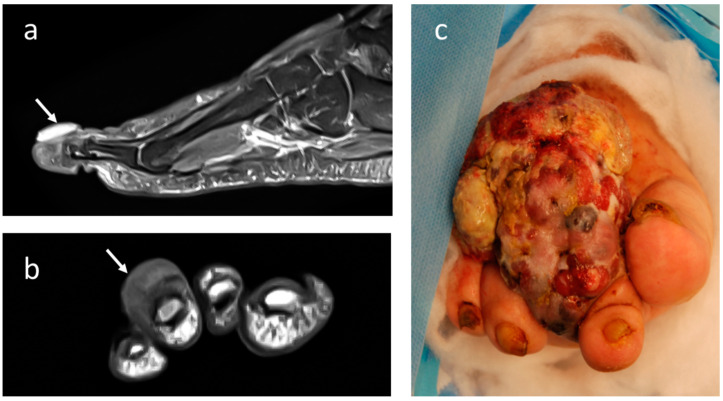
Imaging appearance at T2-weighted MRI with contrast-enhancement in (**a**) sagittal and (**b**) coronal projection of acral melanoma of the 3rd distal foot phalange (small arrows). (**c**) Clinical appearance of large melanoma affecting the foot in a 70-year-old female patient.

Melanoma represents 4% of skin cancers but is extremely lethal, causing 80% of total skin cancer deaths. Approximately 15% of melanomas are located on the foot [62]. Acral melanoma (AM) is a subtype of malignant melanoma found on acral skin, primarily in the skin of the plantar region or in the subungual region (nail beds) [63]. Subungual melanoma accounts for 1–3% of all melanomas diagnosed, mostly affecting the hallux. It affects both men and women [64]. Melanoma is rare in the growth ages, with an incidence that increases with age until the peak between the sixth and eighth decades of life [65] (Figure 4).

AM is the most frequent subtype in populations with darker skin phototypes [63], in the variants (1) acral lentiginous, (2) nodular, and (3) desmoplastic [66].

Chronic or intermittent sun exposure associated with sunburn are the main factors implicated in the genesis of the disease. However, for lesions arising in unexposed areas such as the nail unit and soles of the feet, additional factors may contribute. The role of trauma has been much debated but remains unresolved. Pre-existing plantar lesions can be a risk factor, as suggested by the presence of higher percentages of nevi (junctional or compound) in less sun-exposed sites. Some authors have analyzed exposure to agricultural and industrial chemicals as possible factors associated with tumor development [65].

Melanoma typically presents as a darkly pigmented macule or nodule on the skin, with variegated, blue–black pigment and irregular borders [63]. These lesions may also be amelanotic, i.e., devoid of pigment, appearing lighter in color [65], resulting in diagnostic delays [67]. The Hutchinson sign and dystrophic nail changes are the latest manifestations of advanced disease [64]. Histologically, two different growth modalities can be recognized: radial and vertical. The first involves atypical melanocytes with a lentiginous growth pattern along the basal cell layers. In the second, an infiltrative growth of the spindle cells towards the deeper layers associated with a lymphocytic infiltrate in the dermis is evident. Many efforts have been made to reach an early diagnosis, in consideration of the high prognostic impact, but its identification is often challenging, leading to easy misdiagnosis [63]. Two different acronyms are used to establish suspicion of melanoma: ABCDE (asymmetry, border, color, diameter, evolving) and CUBED (colored, uncertain diagnosis, bleeding, enlargement, delay) [65].

Factors linked to poor prognosis in subungual melanomas are similar to those seen in other sites: tumor stage at diagnosis, ulceration, Breslow depth, and a positive sentinel lymph node biopsy [64]. Excisional biopsy with wide and deep margins is recommended if there is clinical suspicion of AM, while shave procedures should not be used, as they do not allow an accurate pathological assessment of depth.

Surgery represents the standard of care. International guidelines recommend a wide excision with a safety margin of 2 cm in melanomas with Breslow thickness > 2 mm, while one cm is sufficient in thinner ones. Complete lymphadenectomy should be performed in the case of sentinel positive nodes, as it appears to offer improved survival outcomes. Systemic and local treatments other than surgery (including immunotherapy, cryotherapy, and radiotherapy) can be used with contradictory results [63].

### 4.2. Epithelioid Sarcoma

**Figure 5:**
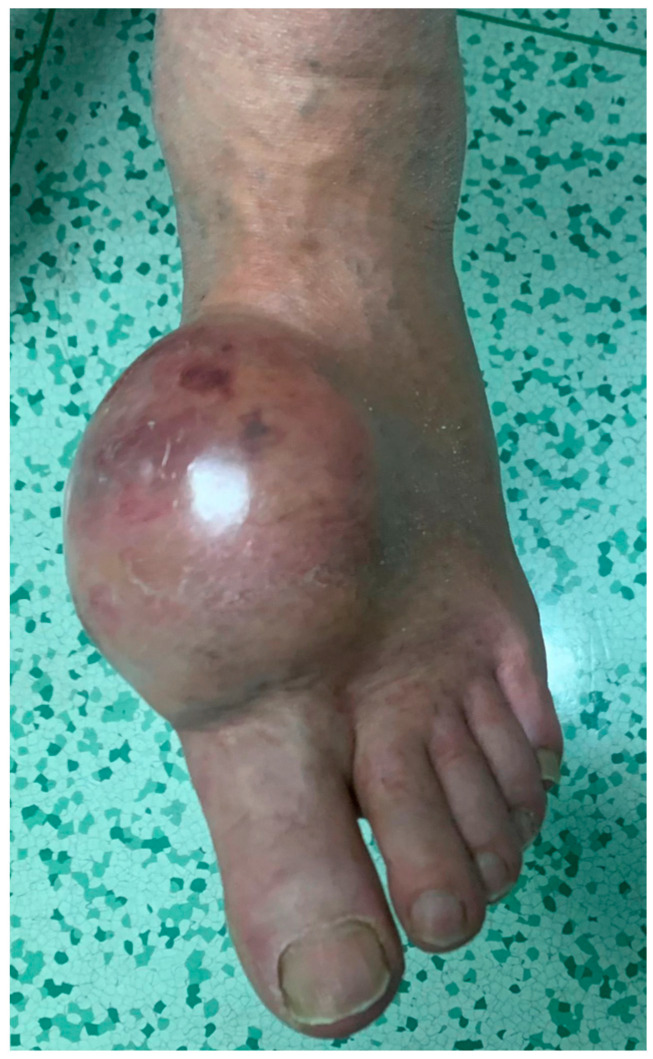
Clinical appearance of a large epithelioid sarcoma of the foot in a 55-year-old female patient.

Epithelioid sarcoma is an uncommon malignancy, representing less than 1% of STS [68]. The classical distal subtype presents in more than 70% of cases, usually in females (2:1) between the second and fourth decades. It typically exhibits an epithelioid morphology [69], with slow growth through subcutaneous layers (Figure 5). At presentation, it manifests as a solitary or multinodular mass with a tendency to ulcerate, placing itself in the differential diagnosis with ulcerated squamous cell carcinomas. Deep-seated tumors are typically larger and firmly attached to tendons and fascia. Because of its uncharacteristic features, misdiagnosis is common [53]. These sarcomas show rare mitotic figures (about 5 mitoses/10 high power fields) but this is generally atypical. Many research studies have reported on epithelioid sarcoma survival rates through the years, ranging from 60% to 80% at 5 years from the primary treatment. However, because the disease is so rare, the numbers vary significantly and metastasis at diagnosis represents an independent prognostic factor. Metastases (involving lung or lymph nodes) develop in 40–50% of cases, usually after local recurrences [70]. The local recurrence rate averages 35% [71].

Surgery is the main treatment if the tumor can be adequately removed with wide, clear margins at first approach. Conventional adjuvant chemotherapy appears to have a limited effect on epithelioid sarcoma. Pazopanib, a multi-tyrosine-kinase inhibitor, has been recently approved in advanced STSs, but its efficacy in epithelioid sarcoma is still unknown [72].

### 4.3. Synovial Sarcoma

Synovial sarcoma is one of the most frequent malignant soft-tissue tumors, accounting for 10% of all soft-tissue sarcomas. It is the most common malignant soft-tissue sarcoma of the foot, accounting for 8% of all primary malignant soft-tissue tumors. Although both sexes are affected, males tend to be affected more often [73]. It can occur at any age, with a peak incidence between 15 to 40 years of age. The etiology remains unclear. Some studies have reported that concomitant trauma might be an accidental event with respect to an actual triggering factor [74]. Clinical features are unspecific: the tumor may appear as a palpable mass, mobile, often painless, or associated with local warmth and discomfort. Often, the lesion has been present for years and might have experienced recent rapid growth. For these reasons, synovial sarcoma can be misdiagnosed as a benign lesion, or other non-oncologic degenerative diseases such as arthritis, bursitis, or synovitis. The authors usually define synovial sarcoma as a “great mimic” due to the wide range of potential differential diagnoses and should always be considered in the presence of unusual soft tissue lesions. Failure to do so can result in the inadequate surgical removal of the tumor without preoperative imaging studies or neoadjuvant treatments [75].

**Figure 6:**
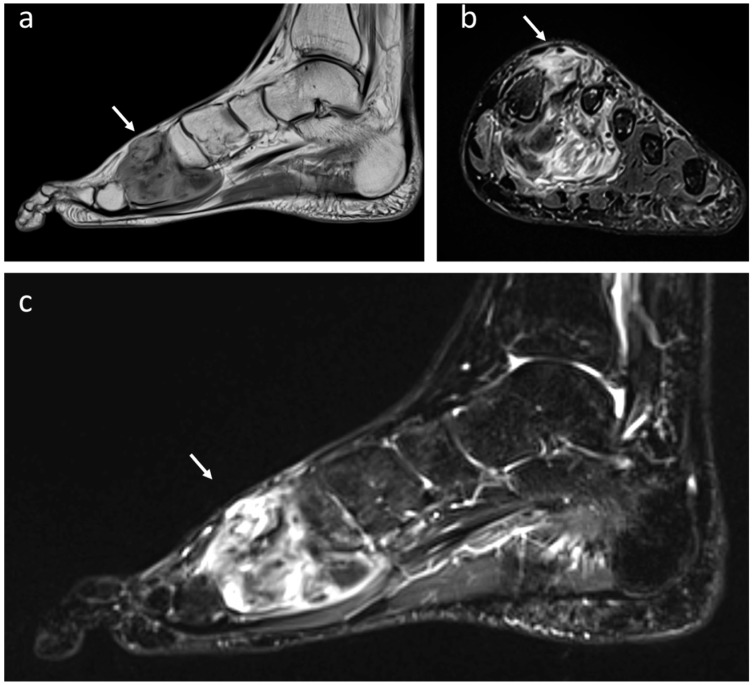
Synovial monophasic Sarcoma of deep foot soft tissues (white arrows) in a 49-year-old male patient: (**a**) T1-weighted MRI showing heterogeneous multiloculated mass isointense to muscle in sagittal projection while an increased signal intensity is shown at T2 STIR in coronal (**b**) and sagittal projection (**c**).

MRI has the advantages of better contrast resolution and soft tissue specificity. The tumor appears heterogeneous and isointense with respect to the muscle on T1-weighted images, with greater signal intensity on T2 [76] (Figure 6).

Synovial sarcomas may calcify in up to 30% of cases, thus mimicking a benign entity [75,76]. Biopsy represents the gold standard. Synovial sarcoma is typically associated with a reciprocal translocation t(x;18) (p11.2; q11.2) [77]. The treatment of synovial sarcoma should be wide or radical excision with adjunctive radiation and chemotherapy. Radiation therapy can be used as an adjuvant strategy when surgical margins are not satisfactory in order to improve local disease control, regardless of the type of surgery planned [75]. Size, margins, grade, and stage have all been found to significantly correlate with survival. Patients treated for tumors smaller than 5 cm have been associated with better survival [78].

### 4.4. Clear-Cell Sarcoma

**Figure 7:**
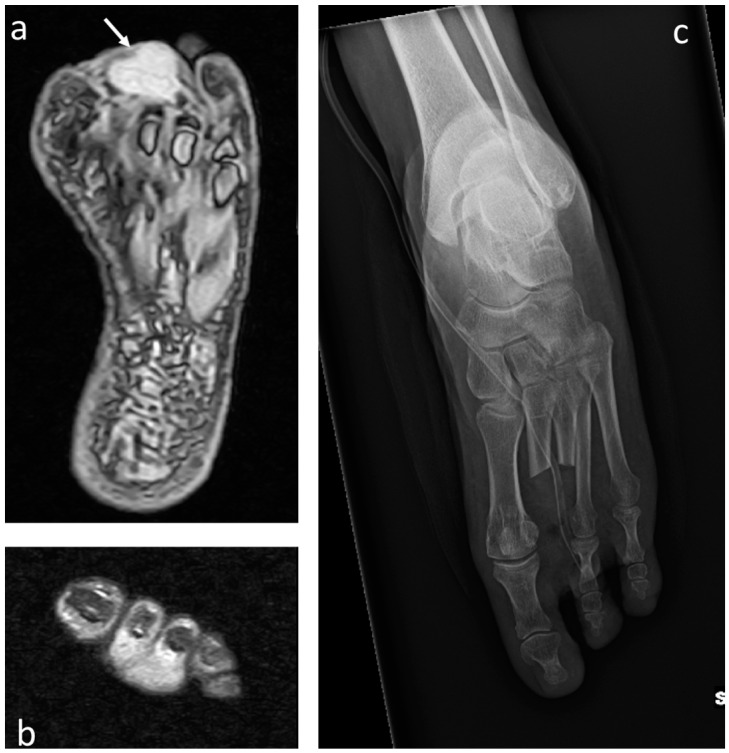
Clear-cell sarcoma of the foot (white arrows) in a 60-year-old female: (**a**) T2 STIR MRI showing the mass with increased signal intensity in axial and (**b**) coronal projection infiltrating the second and third finger. (**c**) Postoperative plain radiograph after double-ray amputation.

Clear-cell sarcoma is a malignant ectoblast tumor that originates from the latent melanin-producing cells that had wandered from the neural crest in the embryonic period [79]. The tumor occurs in patients aged between 20 and 40 years, with female predominance. Different from other histotypes, clear-cell sarcomas account for 33–43% of all the cases in the foot and ankle [80]. Most lesions originate from soft tissues close to tendons, fasciae, and aponeuroses, but occurrence in the superficial dermis has also been reported [81] (Figure 7). They appear as indolent masses, mobile on the underlying floors, without specific pigmentations, characterized by slow growth but with variable dimensions (average diameter 4 cm) [82]. At MRI, the melanin-containing tumors appear hyperintense on T1-weighted images and hypointense on T2-weighted images, with a strong contrast enhancement [83]. The neoplastic cells of clear-cell sarcomas are clear polygonal to fusiform at conventional histologic analysis, with eosinophilic cytoplasm and round nuclei with prominent basophilic nucleoli [84]. Surgical excision is the treatment of choice. Adjuvant chemotherapy shows poor response and is mainly used as a palliative strategy in the late stages of the disease. The 5-year survival rates have been reported to be around 47 to 75%. Smaller tumor volume and early diagnosis are the most favorable prognostic factors for survival [85].

### 4.5. Rhabdomyosarcoma

Rhabdomyosarcomas are a rare STS in adults, but relatively common among patients under 20 years of age (about 4–5% of all cancers in children). Three pathological subtypes may be identified: embryonal rhabdomyosarcoma, alveolar rhabdomyosarcoma, and pleomorphic rhabdomyosarcoma [86]. About 15% of all rhabdomyosarcomas arise in the extremities and they usually have a worse outcome than those occurring elsewhere [87]. It is thought to develop from primitive mesenchymal cells, most likely linked to skeletal muscle embryogenesis. Rhabdomyosarcomas tend to manifest as a low-growth, painless mass arising from deeper soft tissues. The imaging appearance at MRI is common to other STS: iso- to hyperintense on T1-weighted images and heterogeneously hyperintense to muscle on T2-weighted images [88].

Multi-drug chemotherapy regimens significantly improved the survival rates in patients with localized rhabdomyosarcoma over the last decades. When function can be preserved, surgical resection should be considered for local control, but amputation is still used in selected cases. Adjuvant RT can be a very effective treatment modality without resulting in significant morbidity [89].

### 4.6. Leiomyosarcoma

Leiomyosarcoma affects mostly adults and males, with a peak between 40 and 60 years of age. The lower extremities are frequently affected (about 45%), but these STSs can occur almost anywhere because they may originate from cutaneous tissue, soft tissue, and major vessels [90,91]. The cutaneous subtype arises from arrector pili muscles and has a fairly good prognosis. Physical examination generally reveals a soft, painless, palpable mass. MRI may show low signal intensity on T1-weighted sequences, high signal intensity on T2-weighted sequences, and enhancement after Gadolinium injection [92]. Treatment involves wide surgical excision, as patients with inadequate surgical margins are at increased risk for local recurrence and poor prognosis. These lesions respond poorly to adjuvant treatments such as chemotherapy and radiation [93].

### 4.7. Liposarcoma

The 2013 World Health Organization (WHO) identified four main liposarcoma subtypes: myxoid liposarcoma, atypical lipomatous tumor/well-differentiated liposarcoma, dedifferentiated liposarcoma, and pleomorphic liposarcoma. Myxoid liposarcoma represents the most frequent subtype, approximately 5% of all STSs, and about one-third to one-half of all liposarcomas. It primarily affects adults (fifth decade), but is also the most common subtype in the growing ages [94]. Patients’ symptoms include a slow-growing, painless lump deep within the muscle [95]. The classic histology of mixoid liposarcoma consists of small uniform primitive lipoblasts in different stages of maturation, surrounded by a prominent myxoid matrix. High-grade tumors are characterized by sheets of primitive round cells without intervening myxoid stroma and a less obvious vasculature [96].

## 5. Limitations of the Study and Methodology

This is a narrative review with the aim of summarizing the literature on malignant tumors of the foot and describing its current state. The paper is a thorough and critical overview of previously published research on key clinical imaging features and the management of key histologic entities.

This narrative review is based on a selective English and non-English language literature search carried out in PubMed and in the ISI Web of Knowledge database in 2023 using the principal search string ((“bone neoplasms”[MeSH Terms]) OR (“soft tissue neoplasms”[MeSH Terms])) AND (“foot”[MeSH Terms]). Overall, our search string identified 959 articles. Two authors (M.C. and V.L.) independently reviewed the abstracts, while a third author (A.A.) was consulted in case of discrepancies. Articles were divided into different groups according to histologic diagnosis and large case series were separately analyzed. Inclusion criteria were: original articles, English and non-English language, and systemic and narrative reviews. Exclusion criteria were editorials, letters, and case reports. One hundred and twenty-four articles were excluded after reviewing the abstracts, and a further forty-five were excluded following a full examination.

## 6. Conclusions

Although the compact anatomy should facilitate the early detection of tumors of the foot and ankle, early diagnosis is often missed by a lack of awareness on this subject. Vigilance and prudence are key in investigating foot masses, including those with low aggressiveness characteristics. To keep the “diagnostic window” as short as possible, even the experienced orthopedic surgeon and foot specialist needs special training in musculoskeletal oncology and tumor orthopedics. Major oncologic mistakes can be avoided if clinicians take into account all possible differential diagnoses. If a suspicious lump or bump of the foot or ankle cannot be further distinguished by imaging diagnostics, histopathological analysis through image-guided biopsy must be pursued. In the interest of the patient and due to the complexity of this heterogeneous pathology, the expertise of a center for foot and ankle surgery with a specialized tumor surgeon should be consulted or referral to a designated tumor center must be initiated.

## Data Availability

Data available on requests.
